# Pollen Lipids Can Play a Role in Allergic Airway Inflammation

**DOI:** 10.3389/fimmu.2018.02816

**Published:** 2018-12-11

**Authors:** Åslög Dahl

**Affiliations:** Department of Biological and Environmental Sciences, University of Gothenburg, Gothenburg, Sweden

**Keywords:** pollen, allergy, immunomodulatory lipids, lipid-binding allergens, pollen-associated lipid mediators, C1d receptros, iNKT-cells

## Abstract

In seed plants, pollen grains carry the male gametes to female structures. They are frequent in the ambient air, and cause airway inflammation in one out of four persons in the population. This was traditionally attributed to soluble glycoproteins, leaking into the nasal mucosa or the conjunctiva, and able to bind antibodies. It is now more and more recognized that also other immunomodulating compounds are present. Lipids bind to Toll-like and PPARγ receptors belonging to antigen-presenting cells in the mammal immune system, activate invariant Natural Killer T-cells, and are able to induce a Type 2 reaction in effector cells. They may also mimic lipid mediators from mammal mast cells. Pollen grains have a rich lipodome of their own. Among the lipids that have been associated with an atopic reaction are saturated and unsaturated fatty acids, glycophospholipids, sphingolipids, sterols, and oxylipids, as well as lipopolysaccharides from the microbiome on the pollen surface. Lipids can be ligands to allergenic proteins.

About 20–25% of the population suffers from pollen allergy (1), with subsequent reduction of their quality of life and with large societal costs. The risk for sensitization to pollen depends to the degree of exposure. Thus, the main culprits are pollen borne in the air.

The traditional focus has been glycoproteins that are able to bind specific antibodies, quick to be eluted in moisture, before the pollen is eliminated by tears, mucus, or a sneeze. They have been localized to the cytoplasm and to the pollen wall. It is now recognized that also other immunomodulatory compounds emanate from the pollen grains. Among these are lipids, also able to interact with components of the innate immune system. The pollen grain has a lipidome of its own. In this review the main pollen lipids, their location in the pollen grain, and their biological functions are in focus, as well as observed effects on mammal immune reactions.

Lipids are non-polar molecules, which means their ends are not charged, and thus they will be soluble in non-polar solvents rather than in water, which in contrast is polar (2).

## What Is a Pollen Grain?

A pollen grain carries the male gametes to the female reproductive structures. Seed plants have an internal fertilization that evolved in parallel to that found in animals during adaption to terrestrial life. During sexual reproduction, an immotile sperm cell, or in a number of gymnosperms, a spermatozoid, unites with an egg cell inside the tissues of a parental plant, independently of the presence of free-standing water. For this to happen, the pollen grain produces a pollen tube, which acts as a conduit for sperm cells to the egg cell. In seed plants, egg cells are contained within ovules. In gymnosperms, the ovules are born solitary or on the surface of scales often positioned in cones. The pollen grain is delivered at the micropyle, a small opening in the surface of an ovule, which then is penetrated by the pollen tube. In angiosperms, ovules are enclosed in the ovary of the pistil, and therefore, the distance the pollen tube must grow is longer. The pollen grains are generally captured on the stigmatic surface of the pistil, where pollen tubes penetrate into its style.

In contrast to the situation in mammals, the haploid daughter cells from a meiotic division in plants are spores, which divide mitotically as to produce a multicellular, haploid individual that in due course will produce gametes, i.e., is a gametophyte. After gametic fusion, the diploid state is restored. The resulting zygote will divide mitotically and give rise to a multicellular plant, the sporophyte, forming sporangia where the aforementioned meiosis takes place. This alternation between a multicellular diploid and a multicellular haploid phase is called an alternation of generations. In seed plants, gametophytes are dependent on the sporophyte, unisexual, and with few cells. Their entire development takes place within the sporangium.

A pollen grain is a male gametophyte, with an outer wall and coating formed by sporophytic cells. In gymnosperms, microsporangia are usually situated in cone-like structures, whereas in angiosperms, they are identical to the four pollen sacs of the anther. The inside of the sporangium is lined with cells, which provide the microspores with nutrients, and later with wall and coat material. In angiosperms, the first mitosis of the microspore results in a small generative cell, and a much larger vegetative cell, which increases further in size due to an enlargement of the cytoplasm. The generative cell divides mitotically into two sperm cells with large nuclei and comparatively little cytoplasm. This division takes place before or after anther dehiscence and pollen germination, according to species. In gymnosperms, the number of mitotic divisions that produce the male gametophyte typically are two to five. In Taxales pollen (e.g., *Cupressus, Juniperus*), there is no vegetative cell. The generative cell in gymnosperms divides after pollen sac dehiscence and generates two sperm cells or spermatozoids.

## The Mature Pollen Grain and Its Lipidome

### The Vegetative Cell of the Gametophyte

**Figure 1:**
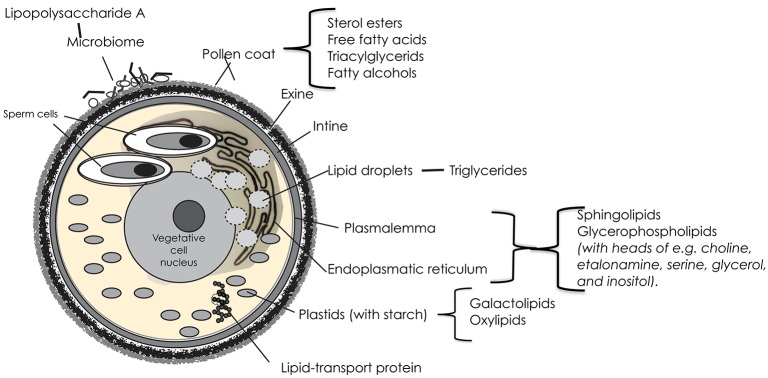
A schematic image of a trinucleate angiosperm pollen grain with the different features that may contribute lipids with a potential impact on allergy; and where the possible candidate lipids are located.

The vegetative cell of angiosperm pollen is the largest one, with a distal plasmalemma, a large central nucleus and a cytoplasm packed with mitochondria, plastids and storage organelles, and surrounding the two sperm cells (Figure 1). The sperms cells have plasmalemmae of their own, and are further enclosed in a plasmalemma from the vegetative cell.

The endoplasmatic reticulum (ER) of the vegetative cell is involved in the synthesis and transport of proteins and lipids. It is extensive, and extends from the plasma membrane surrounding the cell, through the cytoplasm, and forms a continuous connection with the nuclear envelope. The basic units of most of the complex structural lipids of the plasma membrane and the typically large endoplasmatic reticulum are fatty acids, synthesized *de novo* in the chloroplast stroma. In the mature pollen grain, the dominating fatty acids of these structures are octadecadienoic (linoleic; 18:2, *cis*-9,12) and hexadecanoic (palmitic; 16:0) acids (3).

Of the complex structural lipids in membranes, the glycerophospholipids are the most prominent. Their fatty acids are commonly polyunsaturated of C16/C18 type. Glycerophospholipids also usually have an additional group esterified to the phosphate, constituting a polar head group on the lipid molecule. Commonly found head groups in lipids of the pollen membrane are choline, etanolamine, serine, glycerol, and inositol. Because of their bipolarity, the phospholipids in membranes typically form a structure with two layers; hydrophilic heads outwards, hydrophobic fatty acid tails inwards. But in pollen grains, a large portion (40%) of the membrane lipids are non-bilayered, and occur in arrangements appearing as protuberances on the membrane surface (4, 5). Non-bilayer lipids support the dynamic organization of cellular membrane systems and have also been suggested to have an effect on membrane proteins (6, 7).

A stable and chemically resistant layer of sphingolipids protects the outer surface of the bilayered plasma membrane. Sphingolipids compose an estimated ~40% of the total lipids in plasma membrane of plants and are also abundant lipid components of other endomembranes (8). Their backbone is an acylated amino alcohol (a LCB), with fatty acids, generally composed of 14–26 carbon atoms, linked to the amino group. This structure is called a ceramide. It can be modified through changes in chain length, methylation, hydroxylation and/or degree of desaturation of both the alcohol and the fatty acid (9). With further addition of a phosphate or a sugar residue to the alcohol, it can be varied into a plethora of different molecules. The biosynthesis pathway and metabolism of sphingolipids is ubiquitous and highly conserved among eukaryotes, but in plants, they vary more in structure than in other groups (10). Sphingolipids are central to many essential processes in plants including, but not limited to, pollen development. Deficient mutants have been shown to lack a surrounding intine layer (11). They are important in the response to biotic and abiotic stress, such as drought. Presumably, sphingolipids play a role during pollen dehydration. It is likely that stress resistance is due to the fatty acid and long-chain base composition (10).

Glycolylceramides, with headgroup hexoses of either glucose or mannose, are common in the intracellular membranes of pollen grains, relative to leaves. Simple sphingolipid metabolites, such as ceramide and free LCBs, have been shown to be important mediators in signaling cascades involved in various processes such as stress responses, the regulation of cell growth, differentiation, senescence, and apoptosis; in mammals notably also in inflammation. Apart from through the *de novo* pathway, can these metabolites be formed through the hydrolysis of complex sphingolipids. Ceramides and free LBCs can also be phosphorylated through various enzymes (12).

Plastids are double membrane-bound organelles with their own DNA. They originate from proplasts that may differentiate to fulfill various functions, i.e., to be chloroplasts, the site of photosynthesis. In pollen grains, photosynthesis is not necessary, but in the cytoplasm of the vegetative cell, there are usually numerous plastids that accumulate starch. In plastid membranes, galactolipids are more or less dominant. They contain high proportions of the fatty acids octadecadienoic (linoleic; 18:2, *cis*-9,12), octadecatrioneic acid (α-linolenic; 18:3, *cis*-9,12,15) acid, and hexadecatrienoid (roughanic; 16:3 *cis*-7, 10. 13) acid, which under situations of oxidative stress or through enzymatic action can be oxygenated to yield oxylipins (13). Unsaturated fatty acids (in *cis* formation) have “kinks” in their molecular structure, which prevent them from packing as closely as their saturated counterparts. This also makes them susceptible to oxidation (2). In mammals, oxidation catalyzes the generation of oxylipids involved in inflammation. In plants, oxylipins are involved in growth and development, as well as in defense and protection from many abiotic stresses. One example is the phytoprostranes that form from free-radical-catalyzed oxidation to yield oxygen radicals. Singlet oxygen, generated in chloroplasts during photosynthesis, is the most important ROS involved, but also superoxide anion radicals and hydrogen peroxide are involved (14). The result is a chain reaction, leading to the accumulation of hydroperoxides. If they include more than two double bonds, they can be further oxidized to yield many unstable molecules that have a prostaglandin G-ring system and are biologically active. They are powerful gene activators, especially for enzymes involved in the response to challenges by external conditions (15).

### Lipid Droplets

The endoplasmatic reticulum enfolds lipid droplets with a diameter in the range of 0.5–2.0 μm. In pollen grains, they mainly contain triacylglycerides, but also 2–3% phospholipid, probably in a monolayer around the surface of the oil body. The chain lengths of the fatty acids in naturally occurring triglycerides vary, but most contain 16, 18, or 20 carbon atoms. The triacylglycerides accumulate throughout pollen maturation and about 20 % are used during this process (16).

### Lipid-Transferring Proteins

The vegetative cell contains many lipid-binding proteins that contain hydrophobic binding sites for lipid ligands, including triacylglycerols and phospholipids. Most of these proteins will be secreted on the surface of the pollen grain. Although the *in vivo* functions of lipid-binding proteins (LTPs) remain unclear, accumulating evidence suggests a role in the transfer and deposition of monomers required for assembly of waterproof lipid barriers—such as cuticular wax and sporopollenin (17). They are necessary for pollen adhesion and germination on the stigma. Other roles are signaling during pathogen attacks and tolerance to abiotic stresses. LTPs bind both saturated and unsaturated fatty acyl chains. Furthermore, many can fit two fatty acyl chains in their cavity. Some LTPs are reported to bind to hydroxylated acyl chains (18, 19).

### The Intine Is Produced by the Gametophyte

The intine surrounds the vegetative cell, beneath the exine. It consists of fibrillar cellulosa, hemicellulosa and pectine, with associated enzymes that act during germination and pollen tube growth. It develops after the exine. Part of the pectine layer becomes the precursor of the pollen tube wall. The intine is usually thicker under the apertures (20).

In the gymnosperm families Cupressaceae (e.g., *Cupressus* and *Juniperus*), Taxaceae (*Taxus*), and Taxodiaceae (e.g., *Sequoia*), which have inaperturate or monoaperturate pollen, the intine is very thick as compared to the exine and to the diameter of the pollen grain. In contact with water, it swells to rupture, casting off the fragile exine.

### The Outer Wall of the Pollen Grain Is of Sporophytic Origin

#### The Exine

**Figure 2:**
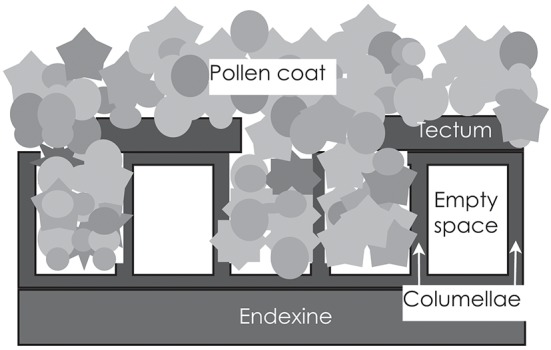
The outer wall of the pollen grain is called the exine, and consists of the very resistant and inert compound sporopollenin. The exine is generally bilayered, with a lower and homogenous endexine and an upper and structured ectexine. The ectexine, in turn, has a foot layer, a layer of rods (columellae) and an outermost tectum. The rods are usually not tightly joined, but are separated by empty space. If the tectum does not cover the lower strata completely (is semitectate), the spaces can be filled with the sticky pollen coat. In insect-pollinated species, the pollen coat is ample and covers the surface of the pollen grain; in wind-pollinated species with intectate pollen, it may fill the spaces between the rods, but be lacking on the surface.

The exine usually consists of two layers [Figure 2; (21)]. The inner one is the endexine, which is a homogenous structure, except for at the apertures. The outer one is the ektexine, which in its complete form has three layers. The lowermost is the foot layer and the outermost is the tectum; the middle layer consists of radially arranged rods (aka columellae), separated by empty space. Variation is large with regard to the thickness, sculpturing and arrangement of the rod layer, as well of the texture and integrity of the tectum. Although consisting of the extremely resistant and complex compound sporopollenin, the exine is not impermeable. Rowley et al. (22) identified numerous radial microchannels, ~25 nm in diameter in mature pollen, that allow for a flow of water and for diffusion of small molecules into spaces between the rods and to the surface coating.

The exine is formed during early microspore life, initially through the contribution of its cytoplasm, but later and above all through accretions by the inner anther wall cell layer, the tapetum, part of the sporophyte.

In most pollen grains, there are thinner parts of the exine, termed apertures, through which the pollen tube generally emerges at germination. They also serve in the accommodation of volume changes at desiccation and hydration (23).

The exine is an extremely stable structure. Its main component is sporopollenin. Its insolubility has been an obstacle to in-depth analysis and its composition and biosynthesis is not fully understood. However, it appears to be a biopolymer of phenylpropanoid and lipidic monomers, such as very long-chained fatty acids and their polyhydroxylated derivatives (24).

#### The Pollen Coat

The outermost layer of the pollen wall, the pollen coat, is of a sticky nature and accumulates in depressions and spaces between the bacula [Figure 2; (25)]. It is mainly composed of lipids and is extremely hydrophobic. In addition, there are also proteins, pigments, aromatic substances and small molecules such as sugars (26). Lipids protect pollen grains against UV light damage, dehydration, and attack by pathogens. They play a key role in pollen-pistil interactions and in pollination by insects or other animals (27). Pollen from anemophilous plants generally contain less of the sticky pollen coat material than those from zoophilous species. This trend is evident in genera where there is a transition from zoophily to anemophily, such as *Fagopyrum* and *Castanea*. In contrast to the case of zoophilous pollen, where there is a sticky layer on the exine surface, it is deposited between the columellae and inside exine cavities in pollen generally dispersed by the wind (28).

Just as the exine, do the contents of the pollen coat mainly emanate from the tapetal cells, after their apoptosis. The coat is formed from their debris (29). The lipid composition of the pollen coat is completely different from that in other parts of the pollen grain.

##### Sterol esters

The relative dominance of sterol esters and fatty acids in the pollen coat appears to vary. Sterol esters contain relatively saturated acyl groups, and may function in the maintenance of the pollen coat in a semi-solid form that enclose and keep the embedded proteins and other substances in place (25). Sterols differ in the length of the side chain and the number and positions of double bonds and attached methyl groups. The sterols found in most tissues of higher land plants mainly consist of sitosterol, stigmasterol, campesterol, and to a lower amount of cholesterol. In pollen the composition is different and more complex. It varies between closely related species, which may be due to variation in pollination system: many pollinating insects lost the ability to synthesize squalene and depend on the uptake from sterols from their nutrition. In a study of pollen from 22 allergenic species (30), β-sitosterol, [(3b-,24R)-ergost- 5-en- 3-yl]oxy], and stigmastan-3,5-diene dominated among sterols, identified in 12–16 pollen species. In addition, stigmasterol and the tripene β-amyrin were present in 6 and 5 species, respectively, of the pollen analyzed. In another study, the major sterol ester in *Brassica napus* was found to be campestdienol, followed by stigmasterol, campesterol and sitosterol (3).

##### Fatty acids

In their study of pollen from 22 allergenic species, Bashir et al. (30) found that saturated and unsaturated fatty acids had the highest concentration and abundance. The dominating polyunsaturated fatty acid was octadecadienoic (linoleic) acid (18:2, *cis*-9,12), followed by octadecatrieonic acid (α-linolenic;18:3, *cis*-9,12,15), but there was a large variation among the species. Among saturated fatty acids, the six most abundant were hexadecanoic (palmitic; 16:0), tetradecanoic (myristic; 14:0), eicosanoic (arachidic; 20: 0), docosanoic (behenic; 22:0), heptadecanoic (margaric; 17:0), and nonanoic (pelargonic; 9:0) acid. Octadecanoic (stearic; 18:0) acid was present in six species, and there were also others with fewer occurrences (30). The ratio between unsaturated and saturated fatty acids varies between species. Both kinds often occur as triacylglycerides in the pollen coat (31).

Very long chain lipids contribute to the hydrophobic cuticle on the surface of all higher plants; their abundance in the pollen coat, however, varies among species. Bashir et al. (30) found 25 different saturated, normal, and branched- chain hydrocarbons with between 6 and 35 carbon atoms. The odd-numbered series of 27, 29, 23, 17, and 21 carbon atoms were the most abundant. Long-chain n-alkanes 31:0 were detected in all grasses, but were rare in other samples. This was also true for 24:0.

Bashir et al. (30) also found significant differences among species with regard to dicarbyxolic acids, with (Z)-butenendioic, butanedioic, and buteneioic (E) acids dominating. They also identified alkenes, fatty alcohols, mono-unsaturated alcohols, and aldehydes.

#### The Pollen Microbiome Contributes to the Lipidome

The sticky pollen coat, containing sugars and lipids, is a favorable habitat for microorganisms. Both bacteria and fungi have been observed on the pollen surface (Figure 1), although the knowledge of their diversity still is quite new. Heydenreich et al. (32) demonstrated that grass pollen grains contained high numbers of Gram- negative and Gram-positive species. Ambika Manirajan (33) found that Proteobacteria was the dominant phylum in all pollen species, followed by Actinobacteria, Acidobacteria, and Firmicutes. Both plant species and pollination type significantly influenced structure and diversity of the pollen microbiota. Variation between species was significantly smaller between insect-pollinated species as compared to the wind-pollinated ones, suggesting an equalizing effect by insect vectors.

The outer membrane of Gram-negative bacteria is dominated by lipopolysaccharide (LPS; aka endotoxin), which is important, e.g., for the structural integrity of the bacteria, resistance to chemical attack, and interaction with predators. LPS can be toxic, due to the component Lipid A, which is a phosphorylated glucosamine disaccharide with multiple fatty acids. Gram- positive bacteria have only one membrane, surrounded by a thick peptidoglycan layer.

## The Pollen Grain in Action

When the pollen grain lands on a stigma in a flower, it must adhere to its surface. The exine structure then plays a significant role, as do compounds in the pollen coat, not least the lipids (34). When they arrive, most pollen grains generally contain little water. In most species, they are partially dehydrated until 15–35 %, just before or after anther opening, and quiescent. A number of species, e.g., of Urticaceae and Poaceae, remain partly hydrated at dispersal (35). In order to fulfill their function, i.e., fertilization, all pollen grains must be rehydrated, germinate and produce a pollen tube.

Stigmas could be “wet” or “dry,” depending on the absence or presence of secretions during the receptive period (36, 37). In species with the wet type of stigma, pollen grains are immediately surrounded by the watery exudates, and are captured due to their stickiness and surface tension (38). In dry stigmas, the surface cells are intact and covered with a cell wall, a waxy cuticle, and a proteinaceous pellicle. Mobilization of pollen coat leads to mixing of lipids and proteins and formation of a “foot” of contact on the stigma surface (39). Thereby, secretion of lipid-rich material is further enhanced, leading to movement of water from the stigmatic papillae to the pollen. This interaction is facilitated by the degradation of lipids by lipases in the pollen coat and pollen wall, as well as by proteins. It leads to swelling of the pollen grain, and the emergence of a pollen tube from one of the apertures. The tube is directed toward the stigma by the gradient of an increasing water potential, which also causes polarization of the pollen cytoplasm. Unsaturated triacylglycerides are required in this process and necessary for the penetration of the stigma (31).

Hydration transforms the non-polar vegetative cell in the pollen grain to be highly polarized within minutes. The sphingolipid glycosyl inositol phosphorylceramides (GPICs) are very common components of plant membranes, but was said to be less abundant in pollen (3). However, in Arabidopsis GPIC-deficient mutants, pollen tubes behaved abnormal, and were not able to properly navigate or communicate with female tissues after pollination, indicating that GPICs indeed are important for the function of the pollen grain. Disruption of sphingolipids containing very long fatty acid chains is known to cause mislocalization of polar membrane proteins (40).

When the pollen grain still is dehydrated, the lipid droplets in the cytoplasm that were not consumed during pollen maturation, are enfolded in a network of the endoplasmatic reticulum. In olive (*Olea europea*) they were observed to enter the emerging pollen tube and to be degraded and apparently be converted directly into membrane lipids (16). In addition, lipid droplets are synthesized throughout the life of the pollen tube, in plasmids present in the vegetative cell cytoplasm that entered the pollen tube. Growth is usually rapid, so the demand for membrane lipids is substantial. It was suggested that sterols also provide necessary membrane material, but rather, they play a role during subcellular processes underlying polar tube tip growth. The composition of sterols changes during pollen tube growth. The change is suggested to promote maximal tube elongation (3).

The *Arabidopsis* alkaline ceramidase TOD1 is a key turgor pressure regulator in plant cells. Turgor pressure plays pivotal roles in the growth and movement of plant cells; thus also in pollen tubes. Li-Yu Chen et al. (41) demonstrate that *Arabidopsis* mutants lacking an alkaline ceramidase have a higher turgor than the wild type and show growth retardation.

## Pollen Lipids in Allergy

When bioaerosols (bacteria, fungi, pollen etc.) come into contact with human tissues facing the external environment, they will meet with so called antigen-presenting cells (APCs), e.g., dendritic cells (DCs). APCs recognize and process antigens and then migrate from the peripheral tissues to a lymph node, where they present the antigen on the cell surface of naive T cells. The dendritic cells also secrete small proteins—cytokines—that influence the polarization of T cell development into different subsets, called effector cells, that induce antibody production and transformation of other cells. The aim is to eliminate the antigen and resolve the infection. After the initial immune response, most of the effector cells die, while a fraction survive and become long-lived memory cells. The host is then said to be sensitized. At future contact with the antigen, he or she will react.

Mature and polarized T cell subsets react to different antigens. Th1 cells react to bacteria, viruses and other intracellular pathogens, and eliminate cancerous cells. In focus of the present review are so called Th2 cells, which have evolved as a response to helminths, but also are activated by allergenic proteins. Dendritic cells stimulate T cells into this pathway through the cytokine IL-4, which in turn also secrete IL-4, as well as IL-13. Thereby, they activate B cells to produce specific IgE-antibodies, They also secrete IL-5, which increases the production of eosinophils in the bone marrow. Eosinophils are lymphocytes containing toxic cytoplasmic granulae. High levels of eosinophils lead to inflammation and tissue damage.

When the sensitized person reencounters an allergen, B-cells proliferate and produce IgE antibodies. These bind to mast cells and cross-link to the allergen, thereby activating the mast cells, which start to release granule proteins, as well as different mediators that provoke the allergic reaction. Mast cells, T cells and macrophages produce cytokines that recruit eosinophils and T cells to the inflammatory site (42).

Natural killer T cells are highly conserved cells, that play an important role at the mucosal surfaces as part of the first line of defense of the immune system. They are present in peripheral tissues and, after activation, display effector functions through the rapid secretion of cytokines, including those involved in a Th2- reaction (43).

Recent discoveries have led to the identification of a novel group of immune cells, the innate lymphoid cells (ILCs). The members of this group are divided into three subpopulations. The subset ILC2s provides a source of the Th2 cytokines, IL-4, IL-5, and IL-13, upon activation by epithelial cell-derived cytokines and lipid mediators (leukotrienes and prostaglandin), and promote structural and immune cell responses in the airways after antigen exposure. Their response appears to be independent of T and B cells (44).

### Antigen-Presenting Cells Carry Lipid-Binding Receptors

Toll-like receptors (TLRs) are a class of proteins that are expressed on the membranes of several cells both in the innate and in the adaptive immune system, as well as of non-immune cells such as in the airway epithelium. TLRs recruit proteins that start a chain reaction, ultimately leading to upregulation or suppression of genes that orchestrate inflammatory responses and other transcriptional events. They recognize molecules that are broadly shared by potentially pathogenic organisms and foreign molecules, which deviate from molecules that belong to the host.

TLR-4 and TLR-2 recognize lipids. TLR-4 recognizes LPS from Gram-negative bacteria, and then interacts with three different extracellular proteins, LPS-binding protein (LBP), CD14 and, myeloid differentiation protein 2 (MD-2). TLR-2 recognizes a wide range of compounds from Gram-positive and Gram-negative bacteria, as well as from mycoplasma and yeast. In both cases, the result is proinflammatory cytokine production (45). Environmental LPS exposure of individuals with allergic airway diseases, including asthma, is known to exacerbate the disease (46).

The peroxisome proliferator-activated receptors (PPARs) are a group of nuclear receptor proteins that are associated with lipids. When the PPAR binds a ligand, transcription of target genes is increased or decreased, depending on the gene. PPARγ is important in asthma, allergy, and airway inflammatory responses through up-regulation of PTEN [phosphatase and tensin homolog; (30)]. Among naturally occurring agents that directly bind with and activate PPARγ are various polyunsaturated fatty acids, which are common in pollen grains, in a free form or as constituents in phospholipids, glycolipids, and triacylglycerol acyl chains, as well as arachnoids, which may occur as oxylipids.

### Lipids Bind to CD1 Molecules

Lipids and glycolipids are recognized by CD1 (cluster of differentiation) molecules, which constitutes a family of glycoproteins, that are expressed on various mammal immune cells and on planar membranes such as in the airway epithelium. They are structurally similar to MHC class I molecules that bind peptids, but are generally not polymorphic.

There are four different CD1 proteins with their own unique way to bind lipids, as their antigen-binding pockets differ. CD1d is expressed on professional APCs including dendritic cells (DCs), macrophages (M), monocytes, and some B cells. CD1d molecules activate a certain subset of NKT cells called “invariant NKT” (iNKT), since they are restricted to CD1d. They were first regarded as a part of the defense against lipids of microbial origin, However, a number of other exogenous and endogenous ligands could be presented by CD1d to iNKT cells, including polar lipids, diacylglycerols, free fatty acids, and triacylglycerols isolated from pollen grains (47, 48).

**Figure 3:**
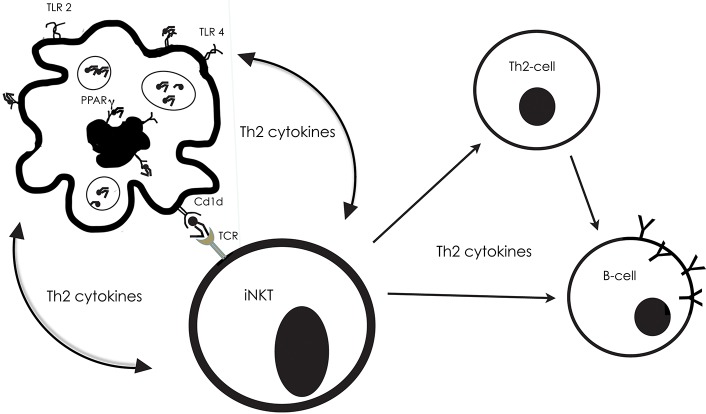
The interplay between antigen-presenting cells (APC), invariant NKT cells, Th2-cells and B-cells, with examples from what is described from reactions to pollen lipids. The Toll-like receptors 2 and 4, on the surface of APCs, as well as the nuclear receptor PPARg in their interior, can bind to lipids, which then are presented by Cd1d molecules on their surface. Then, the Cd1d-bound lipid can be recognized by T cell receptor (TCR) on the surface of the iNKT. Simultaneously, the APC produces cytokines, which skew cytokine production in the iNKT to promote a Th2-inflammatory response. The APC will in turn be stimulated by the iNKT cytokine production, and they will engage in an activation loop, reciprocally stimulating the production of Th2 cytokines (e.g., IL-4, IL-5, IL-13).

Dendritic cells are able to recognize and uptake lipids through their TLR-receptors, or through PPAR. Abos-Gracia et al. (48) exposed monocyte-derived DCs to olive pollen grains in an *in vitro*-culture system (Figure 3). The pollen grain was surrounded and enclosed by the DCs, followed by their maturation and display of an increased CD1d expression, as a result of activation of the PPAR receptor. Abos-Gracia et al. (48) showed that polar lipids from olive pollen (including phospholipids and glycolipids) indeed were able to increase CD1d surface expression. In an earlier study, (49, 50) showed that cloned gδ T lymphocytes from subjects with allergy, but not normal controls, recognized *Cupressus* and *Olea* pollen-derived phosphatidyl- ethanolamine (PE) in a CD1d-restricted fashion. Only 16:0/18:2 and 18:2/18:2 PE were stimulatory. There was no response from disaturated PE, phosphatidylcholine, neutral lipids, or protein extract.

In their assay of lipids occurring on the surface of pollen grains, Bashir et al. (30) stimulated dendritic cells and NKT-cells from two strains of mice with various kinds of lipids that have been related to allergic reactions. DCs and NKTs appeared to engage in an activation loop, reciprocally stimulating the production of Th2 cytokines Figure 3. One of the mouse strains lacked the MyD88 gene, which provides instructions for making a protein involved in signaling within immune cells. The MyD88 protein acts as an adapter, in particular transferring signals from Toll-like receptors and interleukin-1 (IL-1) to the proteins that relay signals inside the cell. The parallel study of cells from wild type and deficient mice would illuminate the importance of these factors for the response to lipid exposure. The results showed that the MyD88 pathway was necessary for the induction of a proinflammatory response to saturated fatty acids, n- alkanes, aliphatic alcohols, and sterols, with some exceptions—and then only in the presence of NKT-cells.

The cytokine IL-13 could induce the secretion of Th2-promoting factors from DCs, further stimulating iNKT-cells to produce more of the Th2-cytokines. The fatty acids eicosanoic acid (20:0) and docosanoic acid (22:0) caused rapid, substantial, and specific up-regulation of Th2-associated IL- 13 from MyD88 deficient DCs/NKT. This result suggested the presence of toll-like receptor (TLR)-independent pattern-recognition receptor in Th2—induction against eicosanoic acid and docosanoic acid. Expression of IL-13 was stimulated also by the n-alkane with 24 carbon atoms and by the aliphatic alcohols 1- tetradecanol and 1-octacosanol (30).

Tumor necrosis factor (TNF, tumor necrosis factor α, TNF α, cachexin, or cachectin) is a cytokine involved in systemic inflammation and is one of those that make up the acute phase reaction. Furthermore, TNF-α might be important in the recruitment of eosinophils to the allergic inflammatory site. Bashir et al. (30) found that the saturated fatty acids 17:0–26:0 induced TNF-α, in DCs from the wild-type mice, with even more enhanced excretion in the presence of NKT-cells. The greatest release was induced by tetracosanoic (24:0) and docosanoic (22:0) acids, followed by hexacosanoic (26:0), and heptadecanoic (17:0) acids. Also n- alkanes, especially with 25 carbon atoms, and aliphatic alcohols, especially 1-tetradecanol, and the sterol b-sitosterol stimulated TNF-α.

Disintegration of pollen may enhance the bioavailability of lipid compounds from inside the pollen grain. As mentioned above, the pollen grains in a number of gymnosperm families (Cupressaceae, Taxaceae, Taxodiaceae) have an exceptionally thick intine and a thin exine. At pollination, the pollen grains are captured in a liquid secreted from the micropyle. The hydrophilic and elastic intine then swells, causing the non-elastic exine to burst and be shed, leaving the intine exposed. After 10–15 min, the expanding intine starts to break, and the cytoplasmic components disperse in the liquid. Exposed acyl side chains of the non-bilayer phospholipids that protrude from membrane surfaces [(5); section The Vegetative Cell of the Gametophyte] may enter the pocket of a CD1 molecule on a DC, making the contact between pollen grain and the DC-cell stable and activating iNKT and other CD1-presenting T cells. Then, DCs migrate into draining lymph nodes, where allergenic proteins are presented to conventional lymphocytes (T- and B-cells), which amplify the allergic reaction (3; 39).

Activation and up-regulation of CD1d by pollen-derived lipids also occur in other types of APCs, such as macrophages and monocytes, apart from in dendriti cells. Both macrophages and monocytes, treated with olive pollen lipids showed an increase in CD1d gene expression (47).

### Lipids Are Ligands to Allergenic Proteins

The allergenic potential of non-specific lipid-binding proteins have been attributed both to their low molecular mass and to their high thermal and proteolytic stability, which allow them to reach the immune system in a biological intact form. But their binding to different types of lipids, including fatty acids, phospholipids, glycolipids, and prostaglandin B, could contribute to the activation of innate immune cells, and enhance an IgE dominant response (51). nsLTP proteins are very versatile with regard to possible ligands. The volume of their internal hydrophobic cavities are sufficient to accommodate either single- or double-chain lipids bound in different modes. It is not yet known how these variants affect the allergenic potency. The possible adjuvant nature of the lipid ligands, when combined with the allergen, must be further evaluated. Lipid-binding to nsLTPs was shown to result in CD1d- restricted activation of iNKT cells promoting allergic sensitization. There might also be other mechanisms, where lipids engage pathogen recognition receptors (PRRs) on the surface of immune and epithelial cells, or influence the absorption of allergens through the epithelial barrier (52).

The major allergen of Brazil nut (*Bertholletia excelsa*), Ber e 1, requires association to a specific fraction of the lipids present in the nuts, in order to trigger allergic Th2 responses in mice and humans (53). This fraction contains neutral and common phospholipids. Whereas the allergen alone is not able to induce sensitization, the complex of allergen and lipid promptly promoted high levels of Ber-specific IgG1 as well as total IgE. It was found that Ber e 1 has a lipid-binding pocket similar to the LTP-binding pocket in wheat protein, and results indicated interaction between the allergen and lipids in the aforementioned fraction. Furthermore, the complex allergen-lipid induced the Th2-associated cytokine IL-4-production by splenic iNKT-cells of mice, leading to the production of anaphylactic antibodies, whereas mice lacking iNKT did not react in this way. Although Ber e 1 is not a pollen-, but a seed-associated allergen, this case illuminates how lipids can enhance the allergenicity of a protein, and the role of iNKT in the provocation of an allergic reaction. Among other components in the active lipid fraction where triclycerides, sterols, and phosphatidylethanolamine, all present in pollen; the latter also reported to be recognized by T-lymphocytes in a Cd1d-restricted fashion in a Olea pollen extract (49).

The natural ligand of the main isoform of the major birch pollen protein Bet v 1 is quercitin, which binds to iron when ligated to the protein. When iron is present, Bet v 1 has no potential to provoke an Th2 response, but is it absent, it manipulates T cells toward a Th2 polarization (54, 55). The ligand interaction increases the volume of the hydrophobic pocket, causing a structural change that might have an impact on the uptake and processing of the protein (56, 57).

### Pollen-Associated Lipid Mediators

Human omega-6 polyunsaturated fatty acid-derived eicosanoids, particularly leukotrienes and prostaglandins, regulate chronic type 2 inflammation on multiple levels, as being released by mast cells, macrophages, DCs, and endo- and epitheliar cells. Upon hydration, pollen grains rapidly release significant amounts of lipids- the so-called pollen-associated lipid mediators (PALMs) that show structural and functional homology to eicosanoids (58). One group is homologous with leukotriene B. They are able to recruit human neutrophils and eosinophils, also in non-sensitized individuals. E1- phytoprostanes are similar to human prostaglandins, sharing the characteristic ring systems. They induce maturing and migration of DCs, and cause them to prime naive T cells to Th2 responses, while they will dampen or inhibit the induction of Th1 responses.

## Conclusion

It is evident that an understanding of atopic reactions to pollen compounds must involve knowledge about the impact of pollen-derived lipids and their ability to activate specialized receptors that start a chain reaction, which “skews” the immune system toward a so called Th2-reaction.

The human nasal mucosa and the conjunctiva can be exposed to potentially allergy-promoting lipids present at the surface of the pollen grain, such as sterols and free fatty acids in the pollen coat, or delivered there from the interior of the grain, such as lipids bound to proteins, and phytoprostranes mimicking mammal lipid mediators. In *Cupressus*, it was shown that hydration of the pollen grain causes disintegration and exposure of membrane-bound phospoholipids, which contribute to allergic reactions. Pollen rupture and exposure of cytoplasmic debris, including possible fragments of ER, plasmalemmas, plastid membranes, and lipid droplets, which are sources of glycophospholipids, sphingolipids, triacylglycerides, and their derivatives, is not unique for cupressacean pollen, but also frequently occurs in that from angiosperms. In contrast to intact pollen grains, these “sub-pollen particles” are as small as to be respirable and may penetrate into the lower airway, where severe allergic reactions including asthma attacks could be triggered (59, 60). Hence, the bioavailability of pollen-derived lipids is substantial and should be further considered.

## Author Contributions

The author confirms being the sole contributor of this work and has approved it for publication.

### Conflict of Interest Statement

The author declares that the research was conducted in the absence of any commercial or financial relationships that could be construed as a potential conflict of interest.
